# Comparing the Extraction Performance in Mouse Plasma of Different Biphasic Methods for Polar and Nonpolar Compounds

**DOI:** 10.1021/acs.jproteome.3c00596

**Published:** 2024-02-06

**Authors:** Friederike Gutmann, Raphaela Fritsche-Guenther, Daniela B. Dias, Jennifer A. Kirwan

**Affiliations:** † Metabolomics Platform, Berlin Institute of Health at Charité − Universitätsmedizin Berlin, Charitéplatz 1, 10117 Berlin, Germany; ‡ Max-Delbrück-Center for Molecular Medicine in the Helmholtz Association (MDC), Robert-Rössle-Straße 10, 13125 Berlin, Germany; § Charité − Universitätsmedizin Berlin, corporate member of Freie Universität Berlin and Humboldt-Universität zu Berlin, Charitéplatz 1, 10117 Berlin, Germany; ∥ Experimental and Clinical Research Center, a cooperation between the Max-Delbrück-Center for Molecular Medicine in the Helmholtz Association and the Charité − Universitätsmedizin Berlin, Lindenberger Weg 80, 13125 Berlin, Germany; ⊥ Berlin Institute of Health at Charité − Universitätsmedizin Berlin, Julius Wolff Institute, Augustenburger Platz 1, 13353 Berlin, Germany

**Keywords:** lipidomics, central carbon metabolism, targeted methods, biphasic extraction, validation

## Abstract

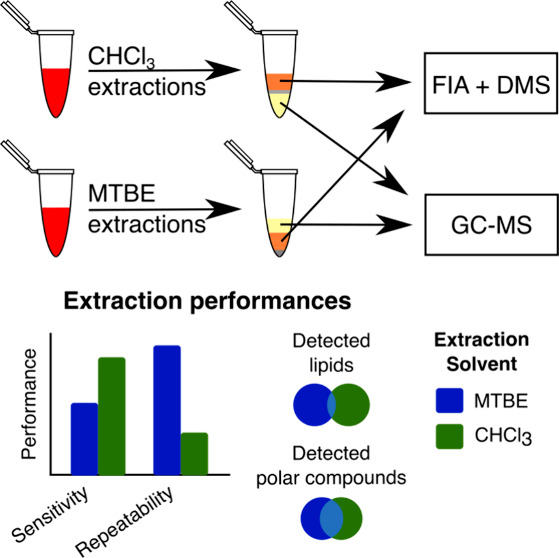

Many metabolomic studies are interested in both polar and nonpolar analyses. However, the available sample volume often precludes multiple separate extractions. Therefore, there are major advantages in performing a biphasic extraction and retaining both phases for subsequent separate analyses. To be successful, such approaches require the method to be robust and repeatable for both phases. Hence, we determined the performance of three extraction protocols, plus two variant versions, using 25 μL of commercially available mouse plasma. The preferred option for nonpolar lipids was a modified diluted version of a method employing methyl *tert*-butyl ether (MTBE) suggested by Matyash and colleagues due to its high repeatability for nonpolar compounds. For polar compounds, the Bligh–Dyer method performs best for sensitivity but with consequentially poorer lipid performance. Overall, the scaled-down version of the MTBE method gave the best overall performance, with high sensitivity for both polar and nonpolar compounds and good repeatability for polar compounds in particular.

## Introduction

The range of metabolites and lipids in a typical biological sample covers a broad spectrum of physicochemical properties. Lipid analysis, in particular, may require very different solvents and analysis methods to extract and optimize the detection of certain lipid classes compared with more polar metabolites. Medical studies, especially those conducted in rodents or which involve repeat sampling, are often sample-limited, reducing the number of individual assays that can be undertaken. Most metabolomics methods require prior extraction of the samples with a solvent mix.^1^ This reduces protein contamination and can also be used to concentrate or dilute the sample, as required. However, each extraction protocol will bias the metabolites that are eventually extracted and detected based on the physicochemical properties of the individual metabolites and solvents employed.^2,3^ Biphasic extraction methods use a combination of different, less miscible solvents at percentages that ensure they form two separate phases. Using biphasic extraction methods to extract a small sample volume, followed by the analysis of both phases, enables a more extensive coverage of the metabolome to be realized and may thus be of great benefit to numerous study designs.^4^

Gas chromatography-mass spectrometry (GC-MS) is a flexible analytical platform that has been shown to provide robust and reproducible results.^5^ Flow injection analysis (FIA) is also widely used for metabolomics analyses and performs well with less volatile lipid species that are unsuitable for analysis with GC-MS. It is particularly beneficial when combined with ion mobility spectrometry to separate isomers and provide additional information on the likely identification of a compound. The SCIEX Lipidyzer platform is a proprietary FIA lipid analysis method that employs a triple quadrupole system coupled with a differential ion mobility spectrometry (DMS) selectivity tool that can profile lipid species of 13 lipid classes.^6,7^ We abbreviated this technology to FIDIMS.

Various extraction protocols are commonly used for metabolomics, often designed around an original technology or use. The Lipidyzer extraction protocol provided by SCIEX specifically for lipids analysis using FIDIMS employs dichloromethane (DCM)/methanol (MeOH) for the extraction and is optimized for lipid recovery. By contrast, the Bligh–Dyer method has been used for polar and lipid analysis and uses a biphasic chloroform (CHCl_3_)/MeOH/water (H_2_O) (2/2/1.8, v/v/v) system.^8,9^ However, using CHCl_3_ as an extraction solvent has disadvantages, not the least of which is its carcinogenic nature. When extracting a biological sample in a biphasic manner with CHCl_3_, a protein and debris layer is formed between the upper polar and the lower nonpolar phase. This leads to potential contamination when recovering the lower phase, as any needle or pipet tip needs to enter through the insoluble interphase. Other extraction solvents with less hazardous and disadvantageous laboratory characteristics have been suggested, but none was as efficient as the previously reported methods, which employed CHCl_3_.^10,11^ However, a promising advancement was made by substituting methyl *tert*-butyl ether (MTBE) for CHCl_3_ in an MTBE/MeOH/H_2_O system.^12,13^ Due to the lower density of the nonpolar phase, which mainly consists of MTBE, it floats on top of the polar phase, leaving the insoluble layer at the bottom of the tube during centrifugation. This allows for a more accessible and less problematic recovery of both phases for the metabolomics researcher and the easier use of liquid handling robots. The original protocol used in our study published by Matyash and colleagues (MTBE/MeOH/H_2_O, 10/3/2.5, v/v/v) was enhanced by Sostare and colleagues, who reported different ratios of MTBE/MeOH/H_2_O (2.6/2.0/2.4, v/v/v) to improve the overall yield and reproducibility when applied on human plasma and urine as well as *Daphnia magna*.^14^ However, this study used a NanoMate direct infusion method and based its conclusions on the peak number and separation ability. While providing an overall profile, this approach has limited ability to distinguish isobaric adducts of different lipid species. By contrast, the SCIEX FIDIMS platform uses FIA coupled with DMS as an orthogonal separation technique, facilitating improved differentiation and quantification of up to a thousand lipid species. Using MTBE instead of the initially proposed DCM has also been reported as feasible for sample extraction for this setup.^15^

We determined which biphasic extraction method best combines lipid and polar metabolite extraction using commercially available mouse plasma. We compared a modified SCIEX Lipidyzer extraction protocol employing MTBE, a modified MTBE method by Matyash et al. (2008), and an adapted protocol of the well-established Bligh–Dyer (1959; BD) method to assess their overall combined performance for polar and nonpolar targeted metabolite analysis.

## Experimental Section

A complete description of the materials and methods is provided in the Supporting Information. All extractions were performed on ice and used 25 μL of commercial mouse plasma. Each extraction method was performed on 5 aliquots of the commercial mouse plasma, so 5 technical replicates of each sample were prepared and measured in random order.

### Biological Sample and Preliminary Experiment

Commercial mouse plasma was extracted in five technical replicates following each protocol as described below. Phase volumes were physically measured, enabling the same percentage of each phase to be analyzed for each extraction protocol.

### Internal Standard Preparation and Standard Procedure

A commercial Lipidyzer internal standard kit (SCIEX, no longer commercially available) was used. 50 lipid species corresponding to 13 lipid classes, namely, ceramides (CER), cholesteryl esters (CE), diacylglycerols (DAG), dihydroceramides (DCER), free fatty acids (FFA), hexosylceramides (HCER), lactosylceramide (LCER), lysophosphatidylcholine (LPC), lysophosphatidylethanolamine (LPE), phosphatidylcholines (PC), phosphatidylethanolamines (PE), sphingomyelins (SM), and triacylglycerols (TAG) were supplied in the kit as internal standards (ISTD). ISTD final volumes used were according to those calculated by the SCIEX Lipidyzer software. Two stock mixes of ISTD, one with MTBE and one with CHCl_3_ as the solvent_,_ were prepared for subsequent use in the extractions.

### Bligh–Dyer Method

This protocol uses a ratio of 6.5/4.5/4.05 v/v/v of CHCl_3_/MeOH/H_2_O, a slight modification of the original ratios reported in the Bligh–Dyer. 25% of the 450 μL of MeOH used in this protocol was used to dilute the plasma. After vortexing, the remaining MeOH, 650 μL of CHCl_3_ including ISTD and 383 μL of H_2_O accounting for the water in the plasma were added. The mix was vortexed, centrifuged, and incubated. 300 μL of the upper polar and 240 μL of the lower nonpolar layer were analyzed.

### Modified Matyash Method Scaled Down

This protocol uses a ratio of 2.6/2.0/2.4 v/v/v of MTBE/MeOH/H_2_O. The plasma was diluted with 102.5 μL MeOH and 10.3 μL H_2_O. After vortexing, 52.3 μL MTBE, including ISTD, were added, and the mix was vortexed again. Next, 82 μL of MTBE and 88.8 μL of H_2_O were added, and the mixture was shaken and incubated. The mixture was centrifuged to collect the protein pellet at the bottom of the tube, and 36 μL of the upper nonpolar and 95 μL of the lower polar phase were used for analysis.

### Modified Matyash Method Diluted

This protocol also uses a ratio of 2.6/2.0/2.4 v/v/v of MTBE/MeOH/H_2_O, but the plasma was diluted to 100 μL using H_2_O before adding 403 μL MeOH. 35.9 μL H_2_O (35.9 μL) was additionally added to account for the overall water ratio. The protocol is the same as the scaled-down protocol except for adding 498.3 μL of MTBE overall, including ISTD, and another 349 μL H_2_O. 140 μL upper nonpolar and 380 μL lower phase were used for the analysis.

### Lipidyzer Methods

The original adapted Lipidyzer method is a two-step process. We wanted to evaluate the effect of one versus two extractions on the final results. We, therefore, extracted metabolites using only one extraction step (Lipidyzer 1×) in addition to the original protocol with two extraction steps (Lipidyzer 2×).

### Modified Lipidyzer Method with One Extraction Step (Lipidyzer 1×)

The plasma was diluted in 600 μL of MTBE, including ISTD and 150 μL of MeOH. After vortexing, incubating, and centrifuging, 300 μL of H_2_O was added to the 750 μL supernatant. After another centrifugation step, 222 μL of the upper nonpolar and 157 μL of the lower polar layer were used for analysis.

### Modified Lipidyzer Method with Two Extraction Steps (Lipidyzer 2×)

In this protocol, the ratios were kept the same as those in the one-extraction step Lipidyzer protocol, but the volumes were adjusted. After the centrifugation, 750 μL of supernatant was removed, and the pellet was subjected to a second extraction step in which 300 μL of MTBE and 100 μL of MeOH were added to the pellet before the mixture was vortexed and centrifuged. 350 μL of the resulting supernatant was then transferred to another tube, and 300 μL of H_2_O was added. After centrifugation, 345 μL of the upper nonpolar and 176 μL of the lower polar layer were used for analysis.

### Gas Chromatography-Mass Spectrometry (GC-MS) Measurement

The extracts were dried, and derivatization was performed using 20 μL of a 40 mg/mL methoxyamine hydrochloride solution in pyridine, incubating the mixture, and adding 80 μL of *N*-methyl-*N*-[trimethylsilyl]trifluoroacetamide (MSTFA) before another incubation. An identification mixture was prepared and derivatized, and an alkane mixture for a reliable retention index calculation was included.^16^ The analysis was performed using an Agilent 7890 gas chromatography system with a VF-5 ms column and a Pegasus HT TOFMS-System coupled to a Gerstell autosampler.

### FIDIMS Platform Measurements

An autosampler from a Shimadzu Nexera X2 UHPLC system was coupled with a QTRAPSystem with SelexION DMS Technology, which was either turned ON or OFF. An FIA setup with an isocratic flow rate of 7 μL/min was used. 50 μL of each reconstituted sample was injected, and 20 spectral scans were collected for each lipid per run. Multiple reaction monitoring (MRM) and positive/negative switching were used to measure lipid species before samples were quantified using the Lipidomics Workflow Manager (LWM). Positive ion mode detected CEs, CERs, DAGs, DCERs, HCERs, LCERs, SMs, and TAGs. Negative ion mode detected FFAs, LPCs, LPEs, PCs, and PEs.

### Data Analysis

**Figure 1:**
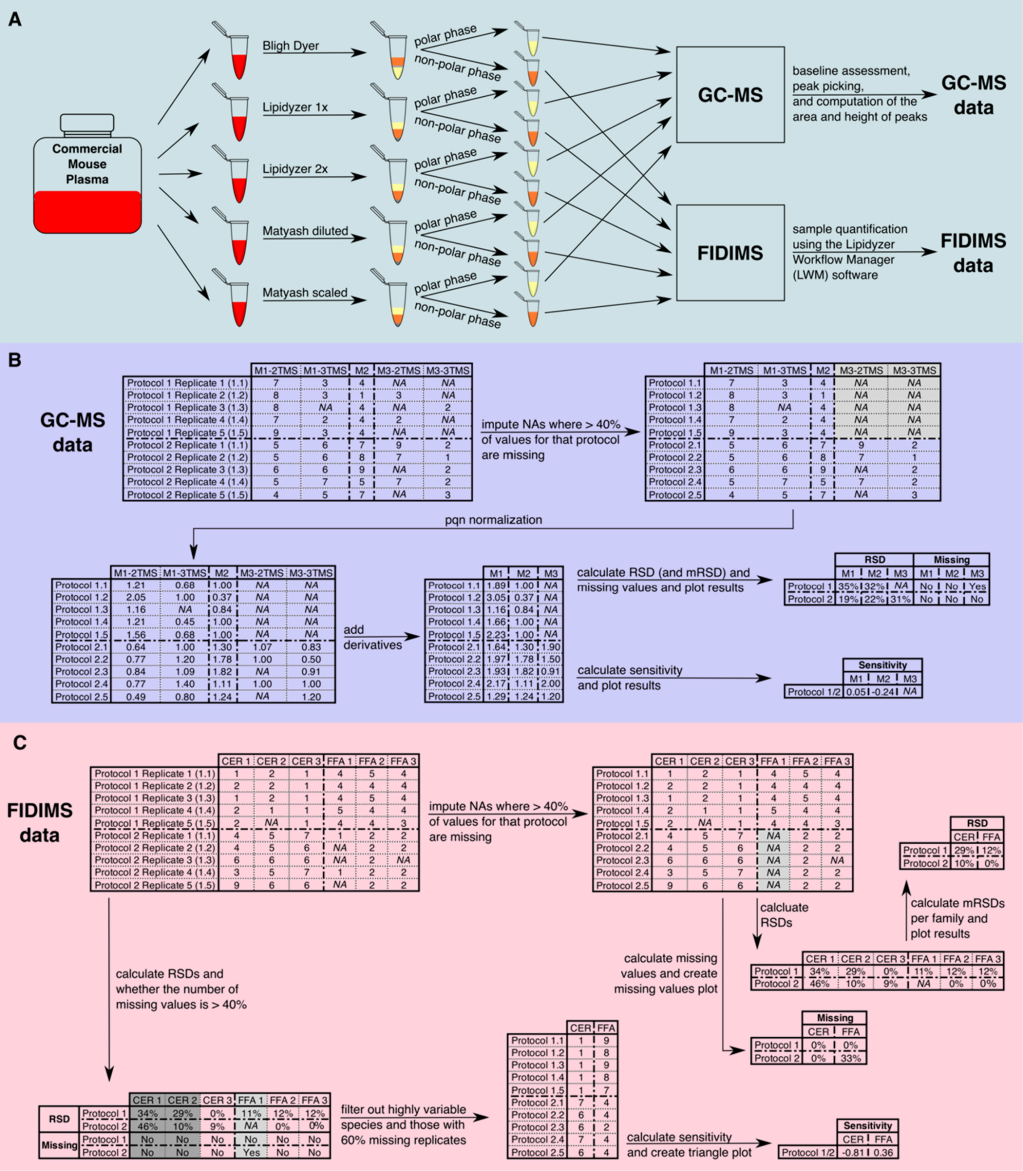
Description of the experimental workflow and filtering regimes. A) Experimental setup and data processing; B) GC-MS data analysis. NAs were imputed for a protocol where the metabolite was missing in at least 60% of replicates. The data were normalized, derivatives were summed up, and the sensitivity and RSDs (plus mRSDs) were calculated; C) FIDIMS data processing and analysis. The data were filtered as described in the methods (global filter, at least 60% values per metabolite and RSD < 15% or per extraction method where NAs were imputed where a lipid species was missing in 60% of replicates). Light gray: Missing value filter—60% of replicates are missing for this species, so either NAs are imputed, or this species is deleted from the data set. Dark gray: RSD filter—the RSD is over 15% for at least one protocol, so this lipid species is deleted from the data set.

Figure 1 summarizes the data processing and analysis. If three or more replicates run on the GC-MS had a missing value for a polar compound, NAs were recorded for all replicates to exclude that compound from further analysis (Figure 1B). The data were normalized using probabilistic quotient normalization (pqn) before derivatives intensities for each metabolite were summed.^17^ Next, as a measure of repeatability, the relative standard deviation (RSD) was calculated for each metabolite, and the median RSD (mRSD) across all metabolites was calculated for each extraction protocol. Missing values were ignored in the calculation of the RSD. The sensitivity of each extraction protocol was calculated as the mean intensity ratio for a compound by one method relative to the measured intensity using the BD protocol.

A lipid species was considered missing in a protocol if at least 60% of replicates analyzed using the FIDIMS platform were missing. RSDs were calculated for each lipid species and summarized as mRSDs per lipid family. To assess sensitivity, we used a filtered data set of lipids that were common to all extraction methods. This data set was created by filtering out lipid species that were missing in >40% of replicates in any individual extraction method and those with an RSD of >15%. The sensitivity of a method was defined as the mean intensity ratio for a compound by one method over the intensity for that compound by one of the other methods. PCs, PEs, and TAGs were divided into four subgroups: short saturated (SS), short unsaturated (SU), long saturated (LS), and long unsaturated (LU).

Where applicable, test results were false discovery rate corrected according to the Benjamini-Hochberg (BH) procedure.

## Results

Five biphasic extraction protocols used in metabolomics were directly compared to evaluate their overall extraction efficiency of mouse plasma: the well-established lab version of the Bligh–Dyer protocol and four protocols employing MTBE instead of CHCl_3_ (Figure 1A). After curation of the GC-MS data, they were processed and analyzed as described in Figure 1B.

### GC-MS Results

First, we analyzed polar metabolites using a GC-MS approach. Specifically, we determined abundances for 62 intermediates corresponding to 45 metabolites of the central carbon metabolism. We detected 23 metabolites in >40% of replicates after extraction with the Bligh–Dyer method, compared to 22 detected metabolites using the diluted Matyash method and 20 after the scaled Matyash version (Supp. Figure 1A). The fewest polar metabolites were detected after the Lipidyzer extraction methods were used, with 18 metabolites detected after Lipidyzer 1× protocol and 17 after the Lipidyzer 2× protocol (Supp. Figure 1A). Methionine and ornithine were only reliably detected using Bligh–Dyer diluted Matyash protocols (Supp. Figure 1A). The Bligh–Dyer extraction is furthermore the only protocol able to extract glyceric acid-3-phosphate and the protocol with the least missing values (Supp. Figure 1A). The Lipidyzer 1× extraction, on the other hand, was the only one that was able to extract isoleucine. The most missing values were produced by the Lipidyzer 2× extraction, which led to only 17 metabolites that could be detected reliably (Supp. Figure 1A).

Repeatability was determined by calculating each metabolite’s relative standard deviation (RSD). All extraction methods achieved mRSDs below a threshold of 15% (Supp. Figure 1B). The scaled Matyash method outperformed all other methods, with an mRSD of 7.4%. Only the Bligh–Dyer method had a comparably low mRSD of 7.6%. Despite these apparent differences in repeatability (Kruskal–Wallis test, *p* = 0.07) none of the achieved mRSDs vary significantly (Dunn’s posthoc test).

Bligh–Dyer outperformed most other methods for sensitivity of polar metabolite detection (*q* ≤ 0.05) (Supp. Figure 1C). Amino acids, particularly the hydrophobic ones, were extracted more efficiently using the Bligh–Dyer method than by any of the other studied methods. Both Matyash-derived methods, especially the diluted Matyash method, performed significantly better than the Lipidyzer methods for most metabolites (Supp. Figure 1C).

### FIDIMS Platform Results

**Figure 2:**
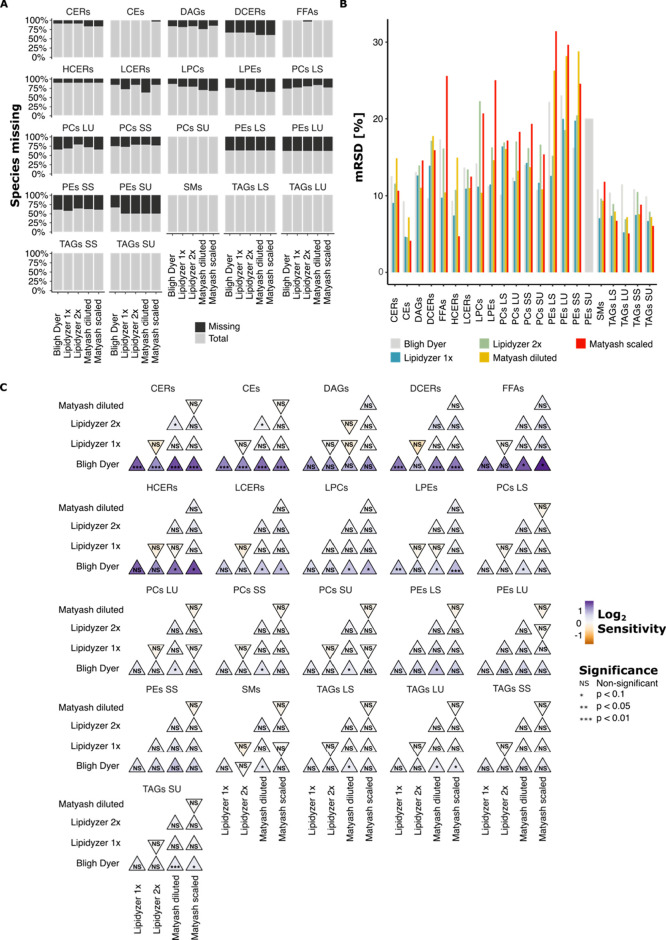
Analysis of the performance of five extraction protocols using the FIDIMS platform. A) Percent of values missing for each lipid family after extraction with each protocol; B) RSDs for each lipid family, calculated based on lipid species within that family, achieved after each extraction; C) sensitivity overview for each lipid family and extraction protocol pair. Sensitivity refers to the mean concentration measured after extraction using one protocol over the concentration measured after extraction with another protocol. To simplify the overview, we logged the sensitivities were logged. Triangles pointing upward indicate a higher mean concentration for the protocol on the *x*-axis than the protocol on the *y*-axis. CE: cholesteryl esters, CER: ceramides, DAG: diacylglycerols, FFA: free fatty acids, LPC: lysophosphatidylcholine, LPEs: lysophosphatidylethanolamine, PC: phosphatidylcholines, PE: phosphatidylethanolamines, SM: sphingomyelins, TAG: triacylglycerols; LS: long saturated, LU: long unsaturated, SS: short saturated, SU: short unsaturated.

Next, we analyzed the nonpolar fraction. For each extraction method, missingness was defined as a failure to be detected in at least 40% of samples. 69 lipid compounds were considered truly missing, as they were not detected in enough replicates of any extraction method. The resulting number of detected lipid species was 727 across the five methods. None of the extraction methods could extract significantly more lipid species of one family compared to the others, although minor differences were seen between methods. Both Matyash methods could detect fewer lipid species reliably than the other three methods, with Lipidyzer 2× (714 detected lipid species) and the Bligh–Dyer method (708) performing best (Supp. Figure 1D). The Lipidyzer 1× extraction protocol performed slightly better (700) than the scaled-down Matyash method (699), which in turn had fewer missing species than its diluted version (697) (Supp. Figure 1D). None of the extraction methods could extract significantly more lipid species of one family compared with the others. The highest percentage of missing values was measured for DCERs and PEs (both 67%). Many LCER (60%), LPE (56%), and LPC (50%) species were also not detected in at least 40% of samples of at least one of the extraction methods. Almost all CEs, FFAs, PCs SU, SMs, and TAGs were detected by all extraction methods (Figure 2A). PEs and HCERs were missing to a comparable extent in all extraction methods, while both Matyash methods had more missing CERs, DCERs, and LPEs and LPCs.

To facilitate a comprehensive repeatability comparison in terms of median RSD (mRSD) at the lipid family level, missing lipid species, as defined above, were filtered out for each extraction method individually. The RSD was calculated per lipid species, and the median RSDs were calculated at the lipid family level. The scaled-down Matyash method stood out with high mRSD values in multiple lipid families, especially in FFAs, LPEs, PEs, and PCs (Figure 2B). These lipid families generally showed high variability in repeatability performance across protocols. The mean of mRSDs per lipid family was below 16% for all lipid families except all PE families. The best performance in terms of repeatability was achieved by the Lipidyzer 1× protocol. Among the MTBE protocols, the Lipidyzer protocol outperformed the Matyash protocol.

Sensitivity analysis revealed a significantly higher sensitivity of the diluted Matyash method compared to the Bligh–Dyer method for all lipid classes (Figure 2C and Supp. Figure 2A). It also outperformed the Lipidyzer 2× extraction method in sensitivity to CERs and CEs. Although compared to the Bligh–Dyer extraction method, all other approaches showed a higher sensitivity for all lipid classes, the FIDIMS extraction methods did not outperform the other methods with the exception of CERs, CEs and DCERs.

### Combined Results

**Figure 3:**
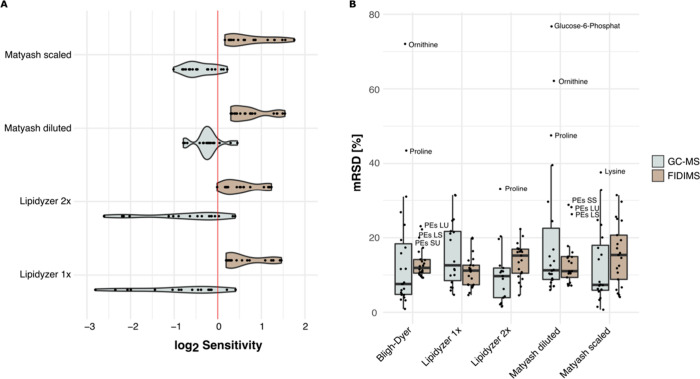
Comparison of the performance of five extraction protocols for polar and nonpolar compounds. A) Extraction efficiency of each protocol depicted as sensitivity as described above; B) repeatability achieved by each extraction protocol for both polar and nonpolar metabolites.

To evaluate the overall performance of each extraction protocol, we combined sensitivity and repeatability results for polar and nonpolar compounds (Figure 3). Principal component analysis (PCA) of all replicates showed broadly two groups of extraction methods: one comprising the Bligh–Dyer and Matyash extraction methods and the other the two Lipidyzer methods (Supp. Figure 2B). As the Bligh–Dyer protocol performed best for GC-MS analysis and worst for FIDIMS platform analysis concerning the sensitivity, we chose this protocol as the baseline for comparison. The extraction efficiency of the diluted Matyash protocol was almost as high as the extraction efficiency of the Bligh–Dyer protocol for polar metabolites and higher for all lipid families (Figure 3A). The scaled-down Matyash protocol achieved similar results but with a higher number of polar metabolites that were not as efficiently extracted by that method as the Bligh–Dyer protocol. Both Lipidyzer extraction protocols performed poorly for polar metabolites but better than other protocols for almost all lipid families, as measured by the number of species detected, sensitivity, and repeatability. By contrast, Bligh–Dyer had one of the highest rates of detection of lipid species but with decreased sensitivity compared to that of other methods. Interestingly, Bligh–Dyer seemed particularly efficient at extracting amino acids, with nearly all compounds in which it outperformed other extraction methods belonging to this class: glycine, lysine, phenylalanine, serine, and threonine (Supp. Figure 1C). Additionally, the alpha-keto acid pyruvic acid and the carboxylic acid glutaric acid were also significantly less efficiently extracted by all extraction methods as compared to the BD method.

The repeatability among nonpolar metabolites was higher than among polar metabolites, which varied more in RSDs (Figure 3B). The outlier glucose-6-phosphate extracted by the diluted Matyash protocol can be explained by one replicate with a significantly higher area in comparison to the other four. On the other hand, proline, ornithine, phenylalanine, and lysine vary greatly among replicates for all extraction methods. This is likely due to the derivatization method. Previous research suggests that the more stable *N*-*tert*-butyldimethylsilyl-*N*-methyltrifluoroacetamide (MTBSTFA) would lead to a higher sensitivity for amino acids in GC-MS analysis. However, due to steric hindrance, the use of MTBSTFA comes with the disadvantage of incomplete derivatization of carbohydrates.^18^ While the Bligh–Dyer protocol showed relatively high repeatability for polar compounds, the median of all lipid family mRSDs was slightly higher than that of the Lipidyzer 1× and diluted Matyash method. However, these two methods had mRSDs for polar metabolites higher than those of all other protocols. The Lipidyzer 2x and scaled-down Matyash methods had the biggest difference between the repeatabilities achieved for polar versus lipid compounds. They both had high repeatability for polar compounds but low repeatability for lipid compounds, with the Bligh–Dyer method showing a slightly lower mRSD for lipid compounds than the scaled-down Matyash method. PEs were considered outliers for the Bligh–Dyer and the diluted Matyash method but generally showed high mRSDs, as shown in Figure 1B.

## Discussion

Optimizing an extraction protocol involves the consideration of multiple parameters, including maximizing the recovery of target metabolites where a targeted method is desired. The quality of an extraction protocol can be assessed by measuring its repeatability and sensitivity. The repeatability of an extraction method is commonly depicted as relative standard deviation (RSD), which adjusts the standard deviation of a compound’s measured quantity in technical replicates by the mean. The sensitivity of an extraction method is a measure of the lowest concentration of a metabolite that it is possible to detect and quantify. It is dependent both on the detection of a compound in the analytical system and on the recovery of a metabolite from a sample. As the same system was used for detection, we assume that any sensitivity changes are entirely due to the extraction process. Recovery depends on multiple parameters, including the absolute and relative solubility of a compound in a specific solvent and how this compares to its solubility in other solvents employed (specifically, how it may divide between the two phases in a biphasic system). Recovery will likely be highest when a metabolite is highly soluble in one of the phases and highly insoluble in the other phase. Recovery may also depend on how other metabolites in the sample may influence the solubility of any individual metabolite, either by the absolute amount of solute to solvent or by physicochemical effects such as altering pH. Here, the relative sensitivity of detection was used as a proxy measurement of metabolite recovery. We have assumed that a higher logged mean quantity of a compound measured equates to a higher sensitivity and recovery overall.

We evaluated the performance of five different protocols employing two different organic solvents, either MTBE or CHCl_3_, in extracting lipids and polar compounds from 25 μL of commercial mouse plasma. We have shown that each tested protocol has specific advantages and drawbacks, and the sensitivity and repeatability achieved by each extraction protocol varies between individual compounds. Therefore, the extraction protocol should be selected according to the researcher’s metabolites of particular interest. However, a few general points can be made.

First, both Lipidyzer protocols are unsuitable for polar metabolite extraction due to their low sensitivity for various polar compounds. This is unsurprising as this protocol was specifically developed to investigate a sample’s lipid content and has a relatively poor ratio of aqueous to nonpolar phase, potentially overwhelming the polar solvent phase’s capacity for complete extraction of all polar metabolites. This may explain why both Lipidyzer methods showed low sensitivities for the polar amino acids serine and threonine and the amino acid lysine that carries a positively charged residue. Additionally, the only protocol leading to the detection of the nonpolar amino acid isoleucine was the Lipidyzer 1× method.^19^ These effects may have also caused the higher number of missing values seen for the Lipidyzer-based extraction protocols. For polar metabolites, the effect was particularly noticeable for the Lipidyzer 2× method, where a few metabolites were detected. This is presumably due to the poor extraction of polar metabolites being amplified by the extra dilution of mixing the two extractions.

Interestingly, the repeatability for polar compounds was not overly influenced by the extraction solvent used. The plasma extraction using MTBE resulted in the polar phase floating above the nonpolar phase with the protein pellet situated on the bottom of the vial. This has several advantages, including better recovery of the protein pellet for protein extraction and easier recovery, with less contamination, of both phases. Our data, however, does not suggest that this improves repeatability. This is true for most of the MTBE-containing protocols that we tested. An extraction with MTBE resulted in higher mRSDs than extraction with CHCl_3_ in 3 out of 4 non-CHCl_3_ employing protocols except for the scaled Matyash protocol that resulted in similar repeatability as the Bligh–Dyer method but with a correspondingly lower number of detected compounds than the Bligh–Dyer method. As the latter is the only protocol with similarly small volumes of solvent used for extraction and chemical analysis as the Bligh–Dyer protocol, the absolute volume may influence the technical repeatability. Large solvent volumes can lead to metabolites being retained on vessel walls as the solvent is dried, thus increasing variability in the recovery. In addition, MTBE evaporates quickly, which may also influence the accuracy of the phase recovery step. Indeed, the evaporation of MTBE has previously been shown to affect the repeatability of chemical analysis using an LC-MS system.^20^ Still, all mRSDs per protocol achieved for polar compounds are below 15% and thus below the threshold for bioanalytical method validation.^21^

The Lipidyzer 2× method includes two extraction steps to capture as many metabolites as possible. This approach indeed leads to the lowest number of missing lipids among all of the tested extraction protocols. Especially the Matyash-based methods struggle to extract some lipid families such as LPCs and LPEs. These lipid families have only one acyl chain linked to a glycerol backbone, increasing their hydrophilicity and thus their solubility. This behavior may lead to losing some of the more amphoteric and polar lipids, especially LPCs and LPEs, to the polar phase and potentially better extraction of LPCs and LPEs using polar solvents such as MeOH.^22^ Also, (D)CERs are problematic for both Matyash protocol versions. Less abundant lipids such as these have previously been shown to be significantly influenced by the applied solvent system.^23^

The Bligh–Dyer method was less sensitive for many lipid families than the MTBE protocols reported before.^13^ This aligns with its strong polar compound extraction efficiency, possibly due to the different polarity indices of MTBE and CHCl_3_, which are 2.5 and 4.1, respectively.^24^ The technical repeatability varied significantly between the lipid families and extraction protocols. The high variability of repeatability performance and the high mRSDs achieved by all protocols for PEs may be due to the large number of missing values for this lipid family. The substantial difference between protocols in repeatably extracting FFAs may be due to the volatility of FFA. The small volume of nonpolar phase available for analysis after extraction with the scaled Matyash protocol and the resulting challenges of pipetting reproducibly while avoiding contamination may be a reason for the low repeatability achieved for lipids by this protocol. However, the low mRSDs per lipid family indicate a strong performance regarding the repeatability of lipid extraction by the Lipidyzer method in general.

Finally, this study was not designed to analyze the difference between solvents, but the solvents were seen as being inherently linked with the protocol in question. How directly switching MTBE for CHCl_3_ in a protocol would change the performance remains a question for further research.

Suppose a scientific project aims to analyze central carbon metabolites in addition to lipids. In that case, one could either perform two separate extractions or choose one of the other tested protocols, depending on the study objective. Suppose a lipid analysis is in the foreground. In that case, a Matyash method can be selected, with the diluted version as the preferred option due to its high repeatability for nonpolar compounds. A sensitive extraction of polar compounds can be achieved best with the Bligh–Dyer method employing CHCl_3_, nonetheless with vast signal losses for lipid compounds. Such disadvantages on the lipid side are not seen for the scaled Matyash method, which has a high sensitivity for both compound classes and good repeatability for polar compounds, in particular. Thus, if an in-depth analysis of lipids is only optional and the main interest of the study lies in polar compounds, then the Bligh–Dyer method is the preferred option. Finally, the scientific question should always guide the choice of the extraction protocol.

## Data Availability

Raw data can be found under MTBLS9172 in the MetaboLights library.^25^

## Supporting Information Available

Figure S1: Analysis of the performance of five extraction protocols using a GC-MS approach. Figure S2: Simplified sensitivity results and multivariate analysis. Table S1: Intermediates measured by GC-MS and the corresponding metabolites. Supplementary experimental section. (PDF)
