# Supplementary material for: Comparing the Extraction Performance in Mouse Plasma of Different Biphasic Methods for Polar and Nonpolar Compounds
Source: J Proteome Res. 2024 Feb 6;23(8):2961–9. doi: 10.1021/acs.jproteome.3c00596 (PMC11301682; doi:10.1021/acs.jproteome.3c00596)
Supplement: Supplementary file 1 — pr3c00596_si_001.pdf [file pr3c00596_si_001.pdf]

Comparing the extraction performance in mouse plasma of different biphasic methods for polar and non-polar compounds

Friederike Gutmann[1,2,3,4], Raphaela Fritsche-Guenther[1], Daniela B. Dias[1,5], Jennifer A. Kirwan[1,2,*]

[1] Metabolomics Platform, Berlin Institute of Health at Charité – Universitätsmedizin Berlin, Charitéplatz 1, 10117 Berlin, Germany

[2] Max-Delbrück-Center for Molecular Medicine in the Helmholtz Association (MDC), Robert-Rössle-Straße 10, 13125 Berlin, Germany

[3] Charité – Universitätsmedizin Berlin, corporate member of Freie Universität Berlin and Humboldt-Universität zu Berlin, Charitéplatz 1, 10117 Berlin, Germany

[4] Experimental and Clinical Research Center, a cooperation between the Max-Delbrück-Center for Molecular Medicine in the Helmholtz Association and the Charité – Universitätsmedizin Berlin, Lindenberger Weg 80, 13125 Berlin, Germany

[5] Berlin Institute of Health at Charité – Universitätsmedizin Berlin, Julius Wolff Institute, Augustenburger Platz 1, 13353 Berlin, Germany

* Corresponding Author, Jennifer.Kirwan@bih-charite.de

TABLE OF CONTENTS:

Figure S1: Analysis of the performance of five extraction protocols using a GC-MS approach. A) Venn diagram depicting the number of (shared) detected polar metabolites; B) Median RSDs (%) achieved after each extraction protocol; C) Sensitivity overview for each significantly differing metabolite (multiple comparison test ≤ 0.05) and extraction protocol as compared to the Bligh-Dyer method. Sensitivity refers to the mean area and concentration measured for polar compounds and lipids, respectively, after extraction using one protocol over the intensity measured after extraction with the Bligh-Dyer protocol. To simplify the overview, the sensitivities were logged. The red line at y=0 indicates no difference in sensitivity between the extraction method in question and the Bligh-Dyer extraction method; D) Venn diagram depicting the number of (shared) detected lipid species.

Figure S2. Simplified sensitivity results and multivariate analysis. A) Simplified heatmap of the sensitivity for each lipid class of each extraction method over the Bligh Dyer method. B) Principle component analysis (PCA) of all extraction methods and all compounds.

Table S1: Intermediates measured by GC-MS and the corresponding metabolites.

Supplementary experimental section.

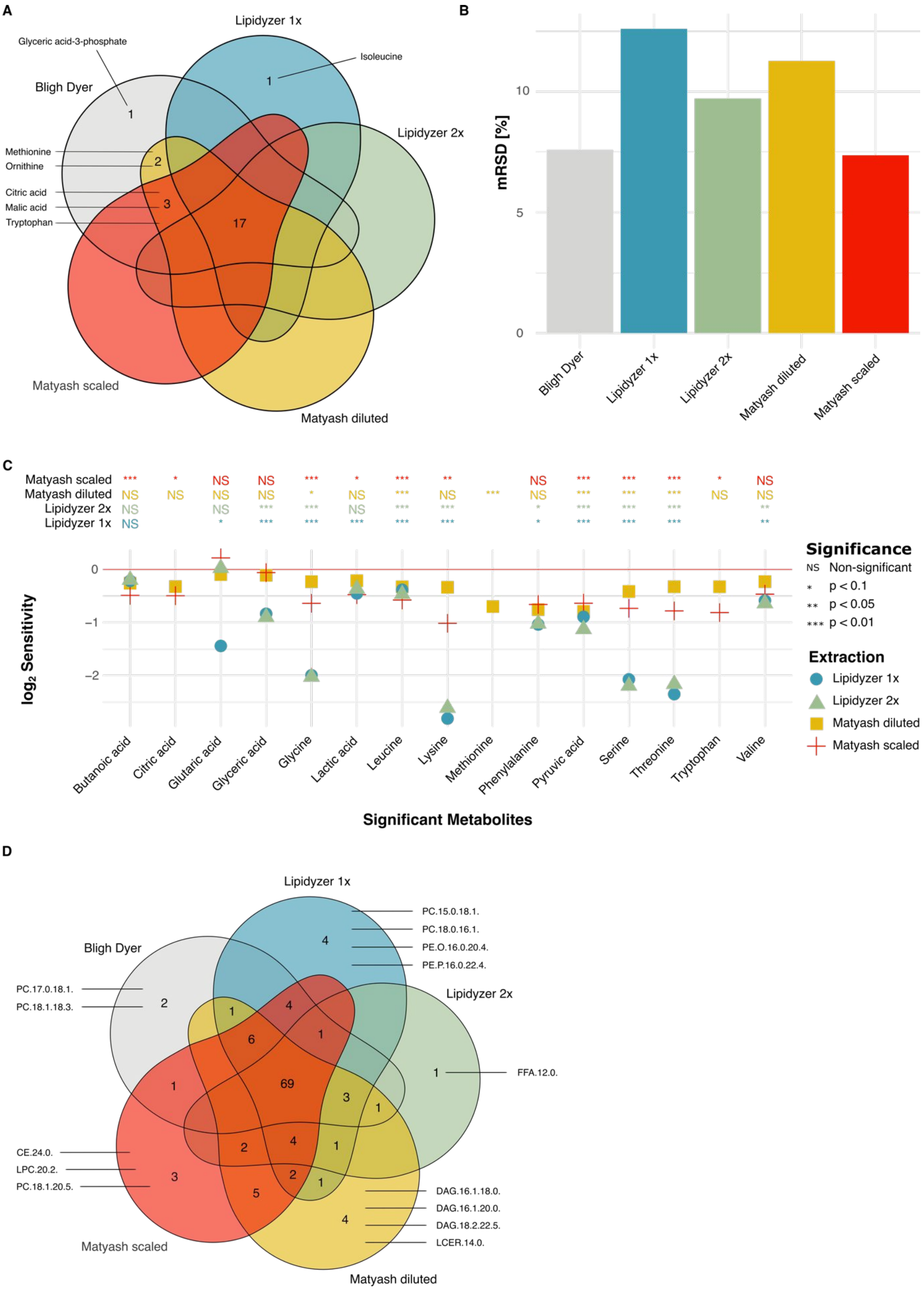

Supp. Figure 1. Analysis of the performance of five extraction protocols using a GC-MS approach. A) Venn diagram depicting the number of (shared) detected polar metabolites; B) Median RSDs (%) achieved after each extraction protocol; C) Sensitivity overview for each significantly differing metabolite (multiple comparison test ≤ 0.05) and extraction protocol as compared to the Bligh-Dyer method. Sensitivity refers to the mean area and concentration measured for polar compounds and lipids, respectively, after extraction using one protocol over the intensity measured after extraction with the Bligh-Dyer

protocol. To simplify the overview, the sensitivities were logged. The red line at y=0 indicates no difference in sensitivity between the extraction method in question and the Bligh-Dyer extraction method; D) Venn diagram depicting the number of (shared) detected lipid species.

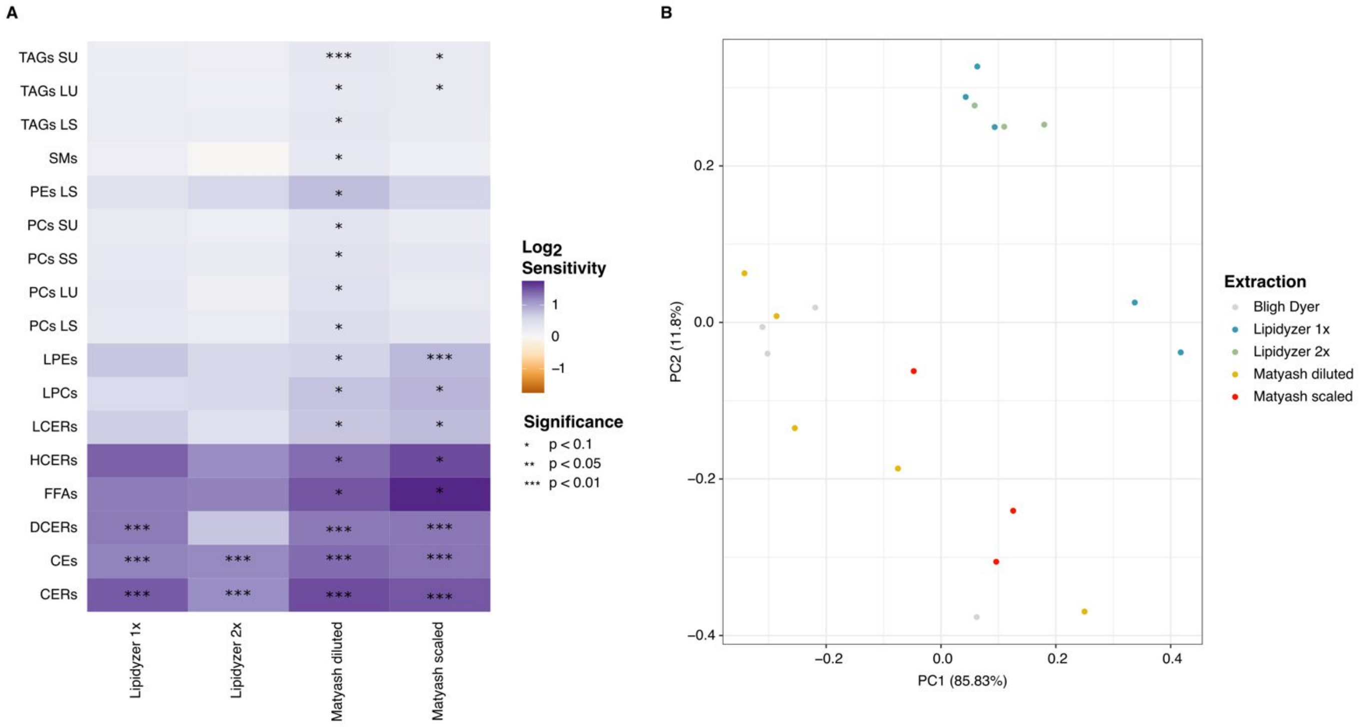

Supp. Figure 2. Simplified sensitivity results and multivariate analysis. A) Simplified heatmap of the sensitivity for each lipid class of each extraction method over the Bligh Dyer method. B) Principle component analysis (PCA) of all extraction methods and all compounds.

SUPPLEMENTARY TABLE

Supp. Table 1. Intermediates measured by GC-MS and the corresponding metabolites.

**Metabolite**	**Derivative**	**Biological group**
Adenine	2TMS	Others (Nucleotide)
Adenosine	3TMS or 4TMS	Others (Nucleobase)
Alanine	2TMS or 3TMS	Amino acid
Asparagine	2TMS	Amino acid
Aspartic acid	2TMS or 3TMS	Amino acid
Butanoic acid, 3-hydroxy	2TMS	Others (SCFA)
Butanoic acid, 4-amino	3TMS	Others (SCFA)
Citric acid	4TMS	TCA
Cysteine	3TMS	Amino acid
Cytosine	2TMS	Others (Nucleobase)
Dihydroxyacetone phosphate	1MeOx 3TMS	Others (Glycerol)
Erythritol	4TMS	Others (Sugar alcohol)
Fructose-6-phosphate	1MeOx 6TMS	Glycolysis
Fumaric acid	2TMS	TCA

Glucose-6-phosphate	1MeOx 6TMS	Glycolysis
Glutamic acid	2TMS or 3TMS	Amino acid
Glutamine	3TMS	Amino acid
Glutaric acid	2TMS	Others (Carboxylic acid)
Glutaric acid, 2-hydroxy	3TMS	TCA
Glutaric acid, 2-oxo	1MeOx 2TMS	TCA
Glyceric acid	3TMS	Others (Glycerol)
Glyceric acid-3-phosphate	4TMS	Glycolysis
Glycerol	3TMS	Others (Glycerol)
Glycine	2TMS or 3TMS	Amino acid
Isobutanoic acid	2TMS or 3TMS	Others (Carboxylic acid)
Isoleucine	1TMS or 2TMS	Amino acid
Lactic acid	2TMS	Glycolysis
Leucine	1TMS or 2TMS	Amino acid
Lysine	3TMS	Amino acid
Malic acid	3TMS	TCA
Methionine	1TMS or 2TMS	Amino acid
Ornithine	3TMS or 4TMS	Amino acid
Phenylalanine	1TMS or 2TMS	Amino acid
Phosphoenolpyruvic acid	3TMS	Glycolysis
Proline	1TMS or 2TMS	Amino acid
Pyruvic acid	1MeOx 1TMS	Glycolysis
Ribose	1MeOx 4TMS	Others (PPP)
Ribose-5-phosphate	1MeOx 5TMS	Others (PPP)
Serine	2TMS or 3TMS or 4TMS	Amino acid
Succinic acid	2TMS	TCA
Threonine	2TMS or 3TMS	Amino acid
Tryptophan	2TMS	Amino acid
Tyrosine	3TMS	Amino acid
Uracil	2TMS	Others (Nucleotide)
Valine	1TMS or 2TMS	Amino acid

MeOX: Methoxyamine hydrochloride. PPP: Pentose phosphate pathway. SCFA: short-chain fatty acid. TCA: Tricarboxylic acid cycle. TMS: Trimethylsilyl derivatives.

SUPPLEMENTARY EXPERIMENTAL SECTION

Biological sample and preparatory experiment. Mouse plasma was purchased at Biotrend Chemikalien GmbH (Cologne, Germany), thawed, and vortexed immediately before analysis. This commercial mouse plasma was extracted in five technical replicates following each protocol as described below. Next, the phase volumes were measured manually. 40% by volume of each phase was taken, of which 40% of this was analyzed, and 40% was kept as a backup aliquot. The remaining 20% allowed for accurate pipetting and ensured no insoluble material in both samples.

Internal standard preparation and standard procedure. A stock solution of internal standards was prepared according to the volumes calculated by the SCIEX Lipidyzer software. A master mix was prepared, and 782 µL was aliquoted as the basis for a stock mix for MTBE employing protocols, while 293 µL was aliquoted for extractions using $CHCl_3$. The larger mixture was resuspended with 600 µL MTBE, and the smaller was mixed with 225 µL $CHCl_3$. A MeOH stock with 6 µg/mL of cinnamic acid was prepared in advance, which was used as an internal standard for the polar analysis. An extract drying step in each protocol ensured that the final ratio of the internal standard to the original plasma ratio was equal for each extraction method, irrespective of the original solvent volume.

For each method, five technical replicates were prepared (Fig. 1A). All samples were extracted one day, dried overnight at 30 °C at 1,550 x g at 0.1 mbar vacuum using a rotational vacuum concentrator (RVC 2-33 CDplus, Christ, Osterode am Harz, Germany), and stored at -20 °C. All solvents and vials were cooled and kept on ice throughout the analysis.

Bligh-Dyer method. 25 µL of the sample was added into prechilled Eppendorf tubes that contained 112.5 µL MeOH. After 15 seconds of vortexing, 337.5 µL MeOH containing 6 µg/mL cinnamic acid, 25 µL Lipidyzer internal standard in $CHCl_3$, and 625 µL $CHCl_3$ was added. Next, 383 µL H_2O was added, and the mixture was vortexed for 15 seconds and left on ice for 10 minutes to separate the phases. Samples were centrifuged at 2,560 x g for 20 minutes at 4°C and left at room temperature for 10 minutes. Two aliquots of 300 µL polar phase and two aliquots of 240 µL non-polar phase were collected in 1.5 µL Eppendorf tubes. The remaining non-polar phase was used for the pooled quality control (QC) samples. A ratio of 6.5/4.5/4.05 of $CHCl_3$/MeOH/H_2O (v/v/v) was used.

Modified Matyash method scaled down. The original modified Matyash protocol uses 100 µL plasma. Our goal was to limit the use of plasma to 25 µL due to the low volume of plasma that can be collected from one mouse. Therefore, we scaled down the entire protocol by 4, keeping all ratios (Matyash scaled). 102.5 µL cold MeOH with 6 µg/mL cinnamic acid was aliquoted in cold Eppendorf tubes. 25 µL sample and 10.3 µL H_2O were added on top. Samples were then vortexed for 15 seconds before adding 25 µl Lipidyzer internal standards in MTBE and 26.3 µL pure MTBE. After vortexing samples for 15 seconds, 82 µL MTBE and 88.8 µL H_2O were added to induce phase separation. Next, samples were shaken in a ThermoMixer® C (Eppendorf, Hamburg, Germany) at 2000 rpm for 1 minute at 4 °C, incubated at 4 °C for 10 minutes, and centrifuged for 10 minutes at 4 °C and 2,560 x g. After standing for 10 minutes at room temperature, two aliquots of 36 µL non-polar and two aliquots of 95 µL polar phase were recovered. The rest of the lower polar layer was used for pooled QC samples. The added volumes equate to a ratio of 2.6/2.0/2.4 for MTBE/MeOH/H_2O (v/v/v).

Modified Matyash method diluted. Since we had scaled down the Modified Matyash method, we wanted to determine whether the extraction efficiency depends on absolute solvent volume effects. In this protocol, solvent ratios were kept the same as the protocol above, including the total added H_2O, but the proportion of plasma to all solvents was altered. 403 µL MeOH with 6 µg/mL cinnamic acid was aliquoted in pre-chilled Eppendorf tubes. 25 µL sample and 110.9 µL H_2O were added before samples were vortexed for 15 seconds. Next, 25 µL Lipidyzer internal standards in MTBE and 176.3 µL pure MTBE were added. Again, the samples were vortexed for 15 seconds, and phase separation was induced by adding 322 µL MTBE and 349 µL H_2O. Samples were shaken at 2000 rpm for 1 minute at 4 °C, incubated at 4 °C for 10 minutes, and centrifuged for 10 minutes at 4 °C and 2,560 x g. Next, samples were equilibrated for 10 minutes at room temperature, and two aliquots of 140 µL non-polar and two aliquots of 380 µL polar phase were aliquoted. The rest of the lower polar layer was used to make a pooled QC sample. Overall, a ratio of 2.6/2.0/2.4 MTBE/MeOH/H_2O (v/v/v) was applied.

Modified Lipidyzer method with one extraction step (Lipidyzer 1x). 575 µL MTBE was added to Eppendorf tubes before 25 µl Lipidyzer internal standard in MTBE was added. Then 25 µL sample was aliquoted on top before 150 µL MeOH with 6 µg/mL cinnamic acid was added to each tube. Samples were vortexed and incubated at 4°C for 30 minutes. The mixture was centrifuged for 5 minutes at 4 °C and 2,560 x g. 750 µL supernatant was transferred to a new Eppendorf tube, and 300 µl H_2O was added. The samples were again centrifuged for 5 minutes at 4 °C and 2,560 x g and left at room temperature for 15 minutes. 222 µL of the upper non-polar layer was aliquoted twice to two Eppendorf tubes. The rest of the upper non-polar layer was used for pooled QC samples. 157 µL of the lower polar layer was transferred twice to two Eppendorf tubes before the rest of the polar layer was captured for pooled QCs. This protocol leads to an MTBE/MeOH/H_2O ratio of 8.48/2.33/3.23 (v/v/v).

Modified Lipidyzer method with two extraction steps (Lipidyzer 2x). This protocol starts like the Lipidyzer protocol above up to and including the centrifugation step. After 750 µL supernatant was transferred to a new tube, however, the left-over matrix in the original tube was extracted once more using 300 µL MTBE and 100 µL MeOH. The tubes were vortexed and centrifuged for 15 minutes at 4 °C and 2,560 x g. 350 µL supernatant from the second extract was combined with the aliquot of supernatant from the first extraction step. Next, 300 µL H_2O was added, and the extracts were again centrifuged for 5 minutes at 4 °C and 2,560 x g. After standing at room temperature for 15 minutes to facilitate phase separation, 2 x 345 µL aliquots from the upper non-polar layer and 2 x 176 µL aliquots from the lower polar layer were transferred to clean Eppendorf tubes. The rest of each layer was used for pooled QCs. The MTBE/MeOH/H_2O ratio used here is the same ratio applied in the modified Lipidyzer protocol with one extraction step.

Gas-chromatography-mass spectrometry (GC-MS) measurement. Analysis of polar extracts followed directly after extraction. The extracts were dried in a rotational vacuum concentrator for one hour to evaporate residual H_2O before derivatization. The extracts were then dissolved in 20 µL of 40 mg/mL methoxyamine hydrochloride solution in pyridine and incubated for 90 min at 30 °C with constant shaking at 800 rpm. Next, 80 µL of N-methyl-N-[trimethylsilyl]trifluoroacetamide (MSTFA) was added, and the mixture was incubated at 37 °C for 60 min with constant shaking at 800 rpm. The extracts were centrifuged for 5 minutes at 18,213 x g, and aliquots of 30 µL were transferred into glass vials for GC-MS measurements. An identification mixture for reliable compound identification was prepared and derivatized similarly, and an alkane mixture for a reliable retention index calculation was included [14]. The metabolite analysis was performed on a Pegasus HT TOFMS-System (LECO Corporation, St. Joseph, MN, USA) complemented with an auto-sampler (Gerstel MPS DualHead with CAS4 injector, Mühlheim an der Ruhr,

Germany). The samples were injected in split mode (split 1:5, injection volume 1 µL) in a temperature-controlled injector with a baffled glass liner (Gerstel, Mühlheim an der Ruhr, Germany). The following temperature program was applied during the sample injection: for 2 min, the column was allowed to equilibrate at 68 °C, then the temperature was increased by 5 °C/min until 120 °C, then by 7 °C/min up to 200 °C, then by 12 °C/min up to a maximum temperature of 320 °C, which was then held for 7.5 min. The gas chromatographic separation was performed on an Agilent 7890 (Agilent Technologies, Santa Clara, CA, USA), equipped with a VF-5 ms column (Agilent Technologies) of 30 m length, 250 µm inner diameter and 0.25 µm film thickness. Helium was used as the carrier gas with a 1.2 mL/min flow rate. The spectra were recorded in a mass range of 60 to 600 m/z with 10 spectra/second. The GC-MS chromatograms were processed with the ChromaTOF software (LECO Corporation, St. Joseph, MN, USA), including baseline assessment, peak picking, and computation of the area and height of peaks without calibration by using an in-house created reference and a library containing the top 3 masses by intensity for 45 metabolites (62 derivatives; see Suppl. Tab. 1) related to the central carbon metabolism. Four samples had to be excluded due to failed injections: two replicates of the scaled-down Matyash method and two of the Lipidyzer 2x method.

SCIEX FIDIMS measurements. Lipids analysis was completed by following the methods recommended by the SCIEX FIDIMS system. This system comprised of a Shimadzu Nexera X2 UHPLC-system autosampler (Shimadzu, Kyoto, Japan) coupled with a QTRAP®System with SelexION® DMS Technology (SCIEX, MA, USA). Two methods were used: one injection with SelexION® Technology ON and another with the SelexION® Technology turned OFF. An FIA setup was employed by introducing samples using the autosampler of a liquid chromatography system (without a column attached) with an isocratic flow rate of 7 µL/min with a ramp-up to 30 µL/min for the last 2 min of the experiment to allow for washing. Data acquisition was around 20 min per sample. 50 µL of each reconstituted sample was injected and analyzed with the recorded peak intensities normalized to the appropriate internal standard. 20 spectral scans were collected for each lipid per run. The lipid molecular species were measured using multiple reaction monitoring (MRM) and positive/negative switching. Positive ion mode detected cholesteryl esters (CE), ceramides (CER), diacylglycerols (DAG), dihydroceramides (DCER), hexosylceramides (HCER), lactosylceramide (LCER), sphingomyelins (SM), and triacylglycerols (TAG). Negative ion mode detected free fatty acids (FFA), lysophosphatidylcholines (LPC), lysophosphatidylethanolamines (LPE), phosphatidylcholines (PC), and phosphatidylethanolamines (PE). In total, the targeted panel can detect up to 1070 lipids. Samples were quantified using the Lipidomics Workflow Manager (LWM). The kits include unlabeled internal standards for the compensation voltage (COV) tuning of the SelexION® device as well as control lyophilized plasma used as a QC sample and QC spike samples (defined lipid amount added to control plasma). Water samples were included to monitor contamination and carryover and solvent blank (mobile phase) for equilibration of the system before analysis. A system suitability test for system performance measurement was carried out as a comprehensive test according to SCIEX instructions before analyzing the samples. Samples are quantified using the LWM software, which reports all the detected lipids in nmol/g.

Data Analysis. All data analysis was conducted in R version 4.0.1. The resulting peak areas from the GC-MS analysis were processed per extraction method individually. If three or more replicates had missing values for a polar metabolite, all values for this metabolite per this extraction method were converted to NAs (Fig. 1B). Firstly, the data were normalized using probabilistic quotient normalization (pqn) before areas of derivatives of each metabolite were added together [15]. Next, RSDs were calculated as described above for each metabolite. Based on these RSDs, the median RSD (mRSD) was calculated for each extraction protocol. This data was also used to calculate principal components after centring and scaling together with FIDIMS-derived lipid family data. The sensitivity of each extraction protocol was calculated relative to the control method, the BD extraction protocol, using equation 1 with n being the number of technical replicates of one protocol in which the metabolite was measured and m being the number of technical replicates of the BD protocol in which the metabolite was measured.

$$Eq.1{:}\ Sensitivity_{Protocol\ 1}^{metabolite\ A} = log_{10}\left(\frac{\frac{1}{n}\sum_{i=1}^{n} area_{Protocol\ 1\ \ i}^{metabolite\ A}}{\frac{1}{m}\sum_{i=1}^{m} area_{Bligh-Dyer\ Protocol_i}^{metabolite\ A}}\right)$$

Whether this difference is significant ($p < 0.05$) was determined using either a Kruskal-Wallis test or a one-way analysis of variance (ANOVA), depending on whether the data were normally distributed, which was determined using a Shapiro test. Multiple comparison test results were then false discovery rate corrected over all metabolites according to the Benjamini-Hochberg (BH) procedure to account for multiple testing. If the multiple comparison test for a metabolite remained significant, either a Tukey test in case of normality or a Dunn test was performed to determine the significance of pairwise group differences. To be particularly strict, posthoc test results were finally BH adjusted over all tested metabolites.

FIDIMS measurement results were filtered and analyzed according to two filtering regimes depending on the analysis outcome required (Fig. 1C). Similar to GC-MS processing, if at least 60% of replicates were missing for one lipid species, it was considered missing in that protocol but not deleted from the entire dataset. The RSD was calculated for each lipid species, and the median RSDs were determined at the family level. The protocol medians were calculated per lipid family, and together with data from GC-MS analysis, these were centred, scaled and used to

perform the PCA. For the analysis of the sensitivity of each method, a different filter regime was applied: lipid species that were missing in > 40% of replicates in any extraction method and those with an RSD of > 15% were entirely filtered out to be able to compare like with like. The mean intensity ratio for a compound by one method over the intensity for that compound by another method was used as a sensitivity measure. To check the significance of differences in lipid detection between extraction methods, either an ANOVA (in case of normality) or a Kruskal-Wallis test was performed. Multiple testing correction was performed following the above-described procedure. Either Tukey or Dunn tests were used to determine group differences, depending on the Shaprio test result. Once more, posthoc test results were BH adjusted over all tested metabolites. The sensitivity was calculated as for Equation 1 but using (sums of) entire lipid families rather than individual compounds. Plots were created using the ggplot2 package (3.3.3) and Inkscape (1.1.1). PCs, PEs, and TAGs were divided into four subgroups: short saturated (SS), short unsaturated (SU), long saturated (LS), and long unsaturated (LU). Decisions on what counted as short chain or saturated varied between lipid species. PCs required at least one chain with a length of between 12 and 16 carbon atoms and at least one fully saturated chain to be considered as SS. If a PC carried at least one short carbon chain, but none of the chains were fully saturated, the PC was called SU. PCs carrying two chains of 17 or more carbon atoms and at least one fully saturated chain were considered PCs LS. All PCs with two unsaturated chains of 17 or more carbon atoms were grouped as PCs LU. The same set of rules applies to PEs. TAGs were considered saturated if they carried no unsaturated chain. TAGs with a total sum of at least 49 carbon atoms were considered long.
